# Genetic diagnosis of congenital hypopituitarism in Turkish patients by a target gene panel: novel pathogenic variants in *GHRHR*, *GLI2*, *LHX4* and *POU1F1* genes

**DOI:** 10.20945/2359-4292-2022-0254

**Published:** 2023-11-10

**Authors:** Tarık Kırkgöz, Semra Gürsoy, Sezer Acar, Özlem Nalbantoğlu, Beyhan Özkaya, Hüseyin Anıl Korkmaz, Filiz Hazan, Behzat Özkan

**Affiliations:** 1 Dr. Behçet Uz Children's Education and Research Hospital Division of Pediatric Endocrinology Izmir Turkey Division of Pediatric Endocrinology, Dr. Behçet Uz Children's Education and Research Hospital, Izmir, Turkey; 2 Dr. Behçet Uz Children's Education and Research Hospital Division of Pediatric Genetics Izmir Turkey Division of Pediatric Genetics, Dr. Behçet Uz Children's Education and Research Hospital, Izmir, Turkey; 3 Dr. Behçet Uz Children's Education and Research Hospital Department of Medical Genetics Izmir Turkey Department of Medical Genetics, Dr. Behçet Uz Children's Education and Research Hospital, Izmir, Turkey

**Keywords:** Pituitary, Hypopituitarism, isolated growth hormone deficiency, combined pituitary hormone deficiency, *GLI2*, *PROP1*, *LHX4*, *POU1F1*

## Abstract

**Objective:**

Congenital hypopituitarism (CH) is a rare disease characterized by one or more hormone deficiencies of the pituitary gland. To date, many genes have been associated with CH. In this study, we identified the allelic variant spectrum of 11 causative genes in Turkish patients with CH.

**Materials and methods:**

This study included 47 patients [21 girls (44.6%) and 26 boys (55.4%)] from 45 families. To identify the genetic etiology, we screened 11 candidate genes associated with CH using next-generation sequencing. To confirm and detect the status of the specific familial variant in relatives, Sanger sequencing was also performed.

**Results:**

We identified 12 possible pathogenic variants in *GHRHR, GH1*, *GLI2, PROP-1*, *POU1F1*, and *LHX4* in 11 patients (23.4%), of which six were novel variants: two in *GHRHR*, two in *POU1F1*, one in *GLI2*, and one in *LHX4*. In all patients, these variants were most frequently found in *GLI2*, followed by *PROP-1* and *GHRHR*.

**Conclusion:**

Genetic causes were determined in only 23.4% of all patients with CH and 63% of molecularly diagnosed patients (7/11) from consanguineous families. Despite advances in genetics, we were unable to identify the genetic etiology of most patients with CH, suggesting the effect of unknown genes or environmental factors. More genetic studies are necessary to understand the etiology of CH.

## INTRODUCTION

The pituitary gland plays a pivotal role in the body's endocrine system by regulating the secretion of most hormones from the other endocrine organs. Congenital hypopituitarism (CH) is defined as the deficiency of one or more hormones secreted by the anterior pituitary (AP) or released from the posterior pituitary (PP) gland.

The fetal development of the pituitary gland is a highly complex process that results from the temporospatial interactions of transcription factors and signaling molecules. Genetic defects involved in this process lead to dysfunction of the pituitary gland, and the clinical presentation of CH varies, ranging from isolated growth hormone deficiency (IGHD) to combined pituitary hormone deficiencies (CPHD). CH can be classified as follows: congenital IGH, defects in growth hormone (GH) production and secretion (*e.g., GH1* and *GHRHR)*, defects in pituitary cell differentiation (*e.g., PROP-1* and *POrU1F1*), and impairment in pituitary development (*e.g., HESX1*, *LHX3, LHX4*, *SOX2, SOX3*, *GLI2*, and *OTX2*) (1).

Often, these genetic defects are accompanied by structural pituitary abnormalities, such as a hypoplastic or absent anterior pituitary gland and/or an ectopic posterior pituitary gland (EPP) or pituitary stalk interruption syndrome, and may be part of a syndrome involving extra-pituitary abnormalities.

CH incidence varies between 1:4,000 and 1:10,000 in live birth (2). It is mostly sporadic, and the etiology can only be identified in a few patients. A limited number of studies and case reports have investigated the genetic etiology of CH in Turkish patients (3–5).

We developed a comprehensive gene panel for screening the previously known pathogenic genes related to CH in Turkish patients with CH, which has not been previously conducted. In this study, to identify the molecular etiology of CH in 47 patients, we screened 11 candidate genes (i.e., *GH1, GHRHR*, *PROP-1, POU1F1*, *HESX1, LHX3*, *LHX4, SOX2*, *SOX3, GLI2*, and *OTX2*) associated with CH using next-generation sequencing (NGS).

## MATERIALS AND METHODS

### Subjects and sample collection

In this study, 47 patients who were followed up at the Pediatric Endocrinology Outpatient Clinic of Dr. Behcet Uz Children Hospital (Izmir, Turkey) with the diagnosis of CH were included. Patients with incomplete medical data and with a previous positive genetic diagnosis of CH were excluded. This study was approved by the local ethics committee considering the Helsinki Declaration (620-202l/15-05), and written informed consent was obtained from the children and their parents before the study.

Patient demographics, body weight and height measurements, pubertal status, and serum basal levels of thyroid-stimulating hormone (TSH), GH, luteinizing hormone (LH), follicle-stimulating hormone (FSH), prolactin (PRL), cortisol, dehydroepiandrosterone sulfate, total thyroxine (T_4_), free T_4_, insulin-like growth factor 1 (IGF-1), IGF-binding protein 3 (IGF-BP3), estradiol, and testosterone were retrieved from the hospital records. The diagnosis of GH deficiency was based on GH peak levels < 7 ng/mL in two stimulation tests (mainly insulin-induced hypoglycemia, L-Dopa, and clonidine tests; glucagon tests were employed within the first years of life) (6). In neonates, GH (cutoff limit < 7 ng/mL) deficiency was evaluated considering the baseline GH measurement during hypoglycemia and IGF-1 and IGFBP-3 levels < −2 standard deviation scores (SDS) for age and sex. The diagnosis of adrenocorticotropic hormone (ACTH) deficiency was based on an extremely low basal cortisol at 8:00 a.m. below 3 µg/dL (83 nmol/L) concomitant with low normal or low ACTH level (<8.3 pg/mL, 1.83 pmol/L) (7). In the case of suspicion of central adrenal insufficiency, low-dose (1 µg) synacthen test was performed for diagnosis, and ACTH stimulation was based on a peak serum cortisol <18 µg/dL (496 nmol/L) (8). TSH deficiency was diagnosed based on a low serum-free T4 with inappropriately low serum TSH levels (<10 mIU). Gonadotropin deficiency was confirmed by low basal FSH, LH, and estradiol/testosterone levels in patients who had already reached pubertal age, but who had a lack of pubertal development (testicles have not enlarged by age 14 in males and thelarche has not appeared by age 13 in females). We used the age- and sex-specific normal ranges of serum prolactin in children developed by Aitkenhead and Heales for defining prolactin deficiency (9). Prolactin levels were measured for at least 3 days. Cranial and pituitary magnetic resonance imaging (MRI) was performed in a follow-up. Height, weight, IGF-1, and IGF-BP3 SDS of the patients were calculated using a child metrics online calculator program (http://www.ceddcozum.com) (10).

### DNA extraction and next-generation sequencing analysis

Blood samples were collected from all included patients and their families who were available and genomic DNA was extracted from leukocytes using the MagPurix kit (Zinexts Life Science Corp., New Taipei City 235, Taiwan), kit according to manufacturer's instructions. For the molecular genetic evaluation, a Custom Target Capture-based Combined Pituitary Hormone Deficiency gene panel (Celemix, Inc., Seoul, Korea), which was designed according to the before-2018 ENMC classification, was used. All coding regions and exon-intron boundaries (±10 bases) of 11 candidate genes including *GH1* (NM_000515/ENST00000323322), *GHRHR* (NM_000823/ENST00000326139), *PROP1* (NM_006261/ENST00000308304), *POU1F1* (NM_001122757/ ENST00000344265), *HESX1* (NM_003865/ENST00000295934), *LHX3* (NM_014564/ENST00000371746), *LHX4* (NM_033343/ENST00000263726), *SOX2* (NM_003106/ENST00000325404), *SOX3* (NM_005634/ENST00000370536), *GLI2* (ENST00000452319), *OTX2* (NM_001270525, NM_021728) were covered. DNA was fragmented for the library preparation. After that, the ends of the DNA fragments were repaired, fragments were A-tailed, adapters were ligated to fragments, indexes were added, probes were hybridized, target libraries were selected with streptavidin beads, and target libraries were amplified, respectively. Samples obtained with the library preparation kit were combined in a single tube at the appropriate concentration. Sequencing reactions were performed with MiniSeq^®^ NGS system (Illumina Inc., San Diego, CA, USA). FASTQ sequencing files were collected and transferred to “SEQ” variant analysis software (Genomize, Istanbul, Turkey). The Integrative Genome Viewer (IGV) (http://software.broadinstitute.org/software/igv/) was used for visualizing the status of each read alignment.

For minor allele frequencies (MAF), possible variants were assessed using the 1000 Genomes Project (https://ftp.ncbi.nih.gov/), Exome Sequencing Project (http://evs.gs.washington.edu/EVS/), and Exome Aggregation Consortium (ExAC, http://exac.broadinstitute.org/). Possible variants that were not presented in Clinvar database (http://www.ncbi.nlm.nih.gov/clinvar/), Human Gene Mutation Database (HGMD, http://www.hgmd.cf.ac.uk) or genetic studies in the published literature were considered as novels and included in the further analysis. Novel variants were evaluated according to 2015 publication of standards and guidelines for the clinical interpretation of sequence variants by the American College of Medical Genetics and Genomics (ACMG) (11). The evaluation also took into account family segregation analysis and outputs from the following web-based bioinformatic tools; Sorting Intolerant from Tolerant (SIFT, http://sift.jcvi.org/), Protein Variation Effect Analyzer (PROVEAN, http://provean.jcvi.org/seq_submit.php), Polymorphism Phenotyping v2 (PolyPhen-2, http://genetics.bwh.harvard.edu/pph2/index.shtml), Mutation Taster (http://www.mutationtaster.org/) software. Sanger sequencing was also performed to detect the status of the familial variants in relatives.

### Statistical analysis

Data obtained from this study were analyzed using GraphPad Prism (statistical software, version 8.0.0). The distribution of data was evaluated with the Kolmogorov-Smirnov test. For numerical comparisons, the independent sample t-test or Mann-Whitney U tests were used for parametric and non-parametric distribution of the measured parameters, as appropriate. Descriptive statistics which were not normally distributed were presented as median and range. Frequency distributions and percentages were given for categorical variables.

## RESULTS

This study included 47 patients (21 girls [44.6%] and 26 boys [55.4%]) from 45 families. The median age at diagnosis of the patients was 5.8 ± 4.9 years (range: 3 months to 15.2 years). The frequency of consanguineous marriage among all patients was 38.7% (18 cases). The perinatal data (i.e., birth weight, birth length, week of gestation, and perinatal complications) were reported by the patients’ attending primary care physicians and parents. Height, weight, and pubertal staging data were obtained using hospital records.

Forty-five patients were affected by growth hormone deficiency (GHD), which was diagnosed in childhood. Furthermore, 17 patients (36.2%) had central hypothyroidism, eight (17.0%) had ACTH deficiency, six (12.7%) had hypogonadotropic hypogonadism, and two (4.2%) had diabetes insipidus. Prolactin deficiency was not diagnosed in any patient. MRI revealed pituitary malformation in 14 patients (seven probands had hypoplastic or absent AP glands with EPP, five probands had isolated EPP, and two probands had hypoplastic or absent AP gland and showed the absence of the bright spot that corresponds to the PP gland). The mean delay in bone age (BA) at diagnosis was 1.7 ± 2.6 years, and the BA/calendar age ratio was 0.65 ± 0.2.

Three patients have a history of breech presentation or other labor and delivery complications. The mean birth weight SDS of the patients was −0.4 ± 1.4. One patient had an isolated cleft palate, another patient had a cleft palate and bilateral syndactyly between the second and third fingers, and another patient had holoprosencephaly. At diagnosis, the height SDS was −2.7 ± 1.5, the weight SDS was −1.7 ± 1.7, the body mass index SDS was 0.1 ± 1.4, and the mean difference between the height SDS and mid-parental height SDS was −1.85. The IGF-1 SDS was −3.0 ± 1.5, and the IGF-BP3 SDS was −1.6 ± 1.7 at diagnosis. The GH peak in the GH stimulation test was 3.0 ± 3.2 ng/mL. The median age at the start of GH replacement treatment was 6.7 ± 4.5 years (range: 3 months to 14 years). All patients received appropriate treatments for their hormonal deficiencies. Various pathogenic variants were detected in 11 patients from 11 families (the overall allelic variant detection rate was 24.4% [11 families/45 families]). The consanguinity rate in variant-positive patients was 63% (7/11). The clinical, laboratory, genetic, and parental characteristics of variant-positive patients with CH are summarized in Table 1.

**Table 1:** Clinical and genetic characteristics of patients with congenital hypopituitarism

Family/patient no	Sex/age of diagnosis (years)	Current Age (years)	Consanguinity	Height SDS	IGF SDS	Hormonal deficiency	Peak GH μg/L	MRI	Gene/zygosity	c.DNA/protein change	Status
1/ I	F/ 11.5	13.4	Yes	-5.4	-2.4	GH	0.4	HAP	GHRHR/(hom)	c.975G>A (p.Trp325Ter)	Novel
2/I	F/3.7	6.2	Yes	-4.3	-4.4	GH	1.2	Normal	GHRHR/(hom)	c.366+1G>A	Novel
3/I	M/2	4	No	0.9	-1.4	GH,T_4_,ADH	0.16	NVPP	GH1/(het)	c.615C>G (p.Ile205Met)	Reported [16]
4/I	F/12	13.9	No	-2.2	-0.5	T_4_, E_2_	12.8	Normal	GLI2/(het)	c.1418G>A (p.Arg473His)	Reported [18]
5/I	M/0.4	11	Yes	-1.8	-2.1	GH	1.0	Normal	GLI2/(het)	c.2440A>T (p.Ser814Cys)	Novel
6/I	F/5.9	15.2	No	-4.1	-2.2	GH	2.6	Normal	GLI2/(het)	c.4558G>A (p.Asp1520Asn)	Reported [28]
7/I	M/1	12	Yes	1.0	-1.3	GH, T_4_, ADH	NA	HAP, NVPP	GLI2/(het)	c.4558G>A (p.Asp1520Asn)	Reported [28]
8/I	F/3.6	16	Yes	-2.3	-2.9	GH,T_4_,E_2_	0.2	Normal	PROP1/(hom)	c.150delA (p.Arg53AspfsTer112)	Reported [20]
9/I	M/5.5	12.5	Yes	-2.2	-2.4	GH,T_4_	0.6	Normal	PROP1/(hom)	c.218G>A (p.Arg73His)	Reported [21],[22]
10/I	M/3.5	9	No	-3.8	-4.4	GH	0.1	Normal	POU1F1/(compound het)	c.54C>G (p.D18E) and, c.892delG	Novel
11/I	F/12	15	Yes	-2.8	-1.4	GH	0.8	Normal	LHX4/(het)	c.288delC (p.Pro98GlnfsTer75)	Novel

HAP: hypoplastic anterior pituitary; NVPP: non-visualized posterior pituitary; NA: not available

The molecular results of the patients included two homozygous *GHRHR* [c.366+1G>A, c.975G>A (p.Trp325Ter)] variants in two patients, one heterozygous *GH1* variant [c.615C>G (p.Ile205Met)] in one patient, three heterozygous *GLI2* variants in four patients in four unrelated families [c.2440A>T (p.Ser814Cys), c.1418G>A (p.R473H), c.4558G>A (p.D1520N)], two homozygous *PROP-1* variants [c.150delA (p.Arg53AspfsTer112), c.218G>A (p.R73H)] in two unrelated families, a compound heterozygous *POU1F1* variant [c.54C>G (p.Asp18Glu) and c.892delG)] in one patient, and one heterozygous *LHX4* variant [novel c.288delC (p.Pro98GlnfsTer75)] in one patient. No significant differences in SDSs of weight, length at birth, GH peak, IGF-1, IGF-BP3, and height were observed between the patients’ positive and negative molecular results (*p* > 0.05). The *in silico* analysis results of six novel variants detected in different genes are presented in Table 2.

**Table 2:** In silico prediction analysis of novel variants

Gene	c.DNA	p.DNA	Exon/intron position	Pathogenicity (ACGM 2015)	Minor Allele Frequency	SIFT	PolyPhen-2	Mutation Taster	PROVEAN
GHRHR	c.366+1G>A		Intron 4	Likely Pathogenic (PVS1, PM2)	Not found	NA	NA	Disease causing	NA
GHRHR	c.975G>A	p.Trp325*	Exon 11	Likely Pathogenic (PVS1, PM2)	Not found	NA	NA	Disease causing	NA
POU1F1	c.54C>G	p.Asp18Glu	Exon 1	VUS (PM2,PP2,BP4)	0.000003778	NA	NA	Disease causing	NA
POU1F1	c.892delG	p.Val272Ter	Exon 6	Pathogenic (PVS1, PM2)	Not Found	NA	NA	Disease causing	NA
GLI2	c.2440A>T	p.Ser814Cys	Exon 13	VUS (PM2, PP3)	Not Found	Damaging	Probably Damaging	Disease causing	Damaging
LHX4	c.288delC	p.Pro98GlnfsTer75	Exon 2	Pathogenic (PVS1, PM2)	Not Found	NA	NA	Disease causing	NA

## DISCUSSION

In this study, we simultaneously screened 11 genes in a cohort of 47 patients with CH using an NGS panel and identified various variants in *GHRHR, GH1*, *GLI2, PROP-1*, *POU1F1*, and *LHX4* in 11 patients (23.4%). In our country, Baş and cols. (4) used a target gene panel including only four genes (*PROP-1, POU1F1*, *LHX3*, and *HESX1*) for the genetic evaluation of patients with CPHD. They demonstrated that *PROP-1* allelic variants, particularly complete gene deletion, were the most common cause of CPHD. In contrast, in this study, which included 11 candidate genes, we showed that *GLI2* was the most common genetic cause of CPHD.

Allelic variants in the *GH1* and *GHRHR* genes are a common cause of IGHD. The *GHRHR* gene encodes the GHRHR protein, which contributes to the release of secretory granules containing GH and GH production through cyclic adenosine monophosphate-dependent transcription. In the literature, studies involving Dutch and Indian individuals reported that the *GHRHR* variant detection rates ranged from 0% to 15% in selected patients with IGHD (12,13). Various types of allelic variant, including nonsense, missense, splice-site allelic variants, micro-deletions, insertions, frameshifts, and regulatory allelic variants, affecting the POU1F1 binding site in the promoter region have been reported (14,15). In this study, a homozygous *GHRHR* variant was detected in two patients born to parents who were first-degree cousins (2/47, 4.3%): c.975G>A (p.Trp325Ter) and c.366+1G>A. These two variants detected in *GHRHR* were evaluated as “likely pathogenic” according to the ACMG 2015 criteria. The c.366+1G>A variant changes the splice-site and protein features, and the c.975G>A (p.Trp325Ter) variant causes a premature termination codon, ultimately leading to nonsense-mediated mRNA decay or truncated GHRHR protein. In contrast, no clear relationship between *GHRHR* gene allelic variants and phenotype has been demonstrated. Classically, mild short stature is expected in *GHRHR* gene allelic variants, whereas cases diagnosed with severe short stature (−6.74 and −8.6 SDS) at a late age have also been reported (16). In our study, the patients with biallelic *GHRHR* variations were diagnosed with IGHD at 3.7 and 5.4 years, and their short stature was moderate (−5.4 and −4.3 SDS). In contrast, MRI revealed anterior pituitary hypoplasia (APH) in a patient with the c.975G>A variant. GHRH and its receptor GHRHR play an important role in the proliferation and function of somatotrophs. APH is frequently reported in cases with homozygous *GHRHR*-inactivating allelic variants (14,17). However, even in patients with the same variant, the clinical phenotype of the pituitary can change, particularly in children. The phenotype difference may be related to the age at which imaging was performed, the experience of the evaluating radiologist, the absence of a standard reference for pituitary dimensions, or the quality of imaging.

*GH1* allelic variants are clinically characterized by short stature, decreased growth rate, and severe growth retardation. In our cohort, we detected a heterozygous *GH1* variant [c.615C>G (p.Ile205Met)], which was reported as affecting GH transduction; the activation of extracellular signal-regulated kinase was reduced to half (18). Our patient, who had the c.615C>G variation in the *GH1* gene, had a history of prolonged labor and hypoglycemia during the infantile period. Additionally, the patient was diagnosed with panhypopituitarism at 2 years of age, and MRI did not reveal the PP gland. We suggest that this variant causes hypoglycemia and prolonged labor and that the birth event affects the PP gland.

*GLI2* is a zinc-finger transcription factor involved in pituitary development, and *GLI2* allelic variants can lead to holoprosencephaly, polydactyly, midfacial, and/or pituitary abnormalities (19). We detected three *GLI2* variants in four unrelated patients, and *GLI2* was the most common genetic cause of CH in our cohort. The patient with the novel variant [c.2440A>T (p.Ser814Cys)] had IGHD and normal pituitary MRI. The c.2440A>T variant is predicted to be a variant of uncertain significance (VUS), and this position is highly conserved. The first patient with the c.1418G>A (p.Arg473His) variant had central hypothyroidism and hypogonadotropic hypogonadism (HH), and the second patient with the same variant had only IGHD. c.1418G>A was identified by França and cols. (20) in a 19-year-old girl with hypopituitarism (i.e., GH, TSH, partial ACTH, FSH, and LH deficiencies) and pituitary hypoplasia.

The *PROP-1* gene has an autosomal recessive manner and is the most frequent cause of CPHD (21). *PROP-1* allelic variants can be associated with variable phenotypes, including deficiencies in GH, TSH, FSH/LH, PRL, and rarely ACTH. In our cohort, we detected two known homozygous *PROP-1* variants. Both patients were born to consanguineous parents. The patient with c.150delA (p.Arg53AspfsTer112) had GHD, TSH deficiency, and HH. This variant was the second most common *PROP-1* allelic variant already described (22). The patient with c.218G>A (p.R73H) had GHD and TSH deficiency, and this allelic variant had been previously reported in patients with GH, TSH, FSH/LH, PRL, and ACTH deficiencies (23,24). In our country, the frequency of *PROP-1* allelic variants was 3.9%-21.8% (estimated frequency, 16.6%) (3–5). In this study, the frequency of *PROP-1* allelic variants was similar to that reported by Kandemir and cols. (3) as 4.2%.

The *POU1F1* gene is a pituitary-specific transcription factor and is associated with the differentiation of thyrotrophs, somatotrophs, and lactotrophs (25). In our cohort, the patient with compound heterozygous variations [c.54C>G and c.892delG)], which are both novel, had IGHD with normal pituitary MRI. However, *POU1F1* allelic variants usually cause pituitary dysplasia, with APH but a normal pituitary stalk and posterior lobe. Sometimes, a normal pituitary gland is detected in young patients and usually turns into APH at older ages (23,24). Chen and cols. (26) hypothesized that the *POU1F1* gene was responsible for pituitary cell survival, and APH occurred progressively in *POU1F1* allelic variants. Our patient with a *POU1F1* allelic variant is now 11 years old, and he will continue to be evaluated in terms of other pituitary hormone deficiencies and the development of APH in the follow-up.

The *LHX4* gene has an autosomal dominant manner and is involved in the control of the development and differentiation of the pituitary gland (27). One patient in our cohort had a novel heterozygous *LHX4* variant (p.Pro98GlnfsTer75), and the patient had only IGHD with normal pituitary MRI. In segregation analyses, the patient's mother had the same variant and short stature (−2.6 SD) with normal IGF-1 levels (117 ng/mL; normal values 100-295 ng/mL) and other pituitary hormone levels. The mother decided not to be investigated further; therefore, no GH stimulation test was performed. This frameshift variant was predicted to be pathogenic, and this position was strongly conserved (phyloP100way = 7.8). In the literature, Cohen and cols. (28) described a 9-year-old boy with IGHD caused by c.194C>T (p.Ala65Val), which he inherited from his mother. Furthermore, Gucev and cols. (29) described a 14-year-old boy with IGHD and myopathy, caused by c.250C>T (p.R84C) variation in *LHX4*. Generally, patients who carry allelic variants with a total loss of function (LOF) in *LHX4* show CPHD and rarely carry partial LOF in IGHD (28). Interestingly, although our patient had a null allelic variant, we detected only IGHD but not CPHD. This finding indicates that the genotype-phenotype relationship is more complex in *LHX4,* and more data are necessary to elucidate the phenotypic spectrum caused by *LHX4* allelic variants.

This study has some limitations. Our study was single-centered, and the number of patients included was relatively low. *In vitro* functional studies are essential to prove the disease-causing effects of novel variants that were interpreted to be as pathogenic/likely pathogenic/VUS by various bioinformatic tools. Additionally, all genetic causes associated with CPHD could not be investigated. Finally, large copy numbers or deep intronic variants could not be analyzed.

In conclusion, we identified various variants in *GHRHR, GH1*, *GLI2, PROP-1*, *POU1F1*, and *LHX4* in 11 patients (23.4%), with 63% molecularly diagnosed in patients from consanguineous families in line with the literature data. We identified six novel variants: two in *GHRHR*, two in *POU1F1*, one in *GLI2*, and one in *LHX4*, expanding the allelic variant spectrum of these genes. However, we could not detect any genetic cause in most patients, suggesting that further studies are necessary to understand the participation of other genetic, epigenetic phenomena and/or environmental factors in the etiology of CH.
